# Combined bilateral lateral rectus recession and medial rectus Faden procedure in true divergence excess exotropia: a case report

**DOI:** 10.1093/jscr/rjaf868

**Published:** 2025-11-05

**Authors:** Ahmed Swelam, Khaled AbdelKhalique abdelshafi Ghaith, Mahmoud Hegab, Sherin Ghaith, Abdulmajeed Al khathami, Selma Milisic

**Affiliations:** Pediatric Ophthalmology & Squint Unit, Magrabi Eye Center – Prince Sultan Street, North Jeddah 23437, Makkah, Saudi Arabia; Department of Ophthalmology, King Fahad Hospital, Al Baha Health Cluster, Abad Algadaae Street, Al Baha 65527, Saudi Arabia; Ophthalmology Department, Faculty of Medicine, Cairo University, 84 Wadi ElNile Street, Al Zaiton, Cairo 11734, Egypt; Al Baha Health Cluster, Ashetebah, Baljurishi, Al Baha 65652, Saudi Arabia; Department of Ophthalmology, King Fahad Hospital, Al Baha Health Cluster, Abad Algadaae Street, Al Baha 65527, Saudi Arabia; Department of Ophthalmology, Public Institution Health Center of Sarajevo Canton, Vrazova 11, Sarajevo 71000, Bosnia and Herzegovina

**Keywords:** divergence excess, intermittent exotropia, AC/A ratio, Faden procedure, bilateral lateral rectus recession

## Abstract

True divergence excess, a subtype of intermittent exotropia, presents with a greater exodeviation at distance than at nearby. Its management often requires tailored surgical strategies to address both distance and near deviations. A 9-year-old Saudi girl presented with intermittent right-eye exotropia. Prism and alternate cover test measurements revealed 30 prism diopters (PD) of exodeviation at distance and 12 PD at nearby. Cycloplegic refraction showed mild hyperopia. The accommodative convergence to accommodation ratio was within normal limits. No significant ocular or systemic comorbidities were present. The patient underwent bilateral lateral rectus recession (7 mm) to correct the distance deviation, combined with bilateral medial rectus Faden procedures to control near deviation. The surgery proceeded without complication. On postoperative Day 1, the patient achieved orthophoria at both distance and near. At the 3- and 6-month follow-ups, ocular alignment remained stable, with only a mild residual exotropia of 5 PD at distance without glasses. No diplopia or motility restrictions were reported. A combined surgical approach of bilateral lateral rectus recession and medial rectus Faden procedure can effectively manage true divergence excess in intermittent exotropia, yielding stable postoperative alignment. Individualized surgical planning remains essential for optimizing outcomes.

## Introduction

Intermittent exotropia (IXT), the most prevalent type of childhood exotropia, affects about 32 out of every 100 000 children under the age of 19 [1]. It is characterized by periods of ocular alignment interspersed with intermittent exodeviation of one eye, which can make precise measurement difficult [2]. One subtype of IXT, divergence excess (DE), is characterized by a significantly greater exodeviation at distance than at near. True DE is frequently linked to a high accommodative convergence to accommodation (AC/A) ratio [3]. This case report presents the surgical management of a 9-year-old patient with true DE, focusing on a tailored approach using bilateral lateral rectus recession and medial rectus Faden procedure to address the distance and near deviations.

## Case presentation

A 9-year-old Saudi female, presented to the clinic with a chief complaint of intermittent outward deviation of her right eye, which was first noted by her mother. The mother could not specify the duration, frequency, or severity of the eye turn. The patient had no significant past ocular or medical history.

### Initial examination

**Figure 1:**
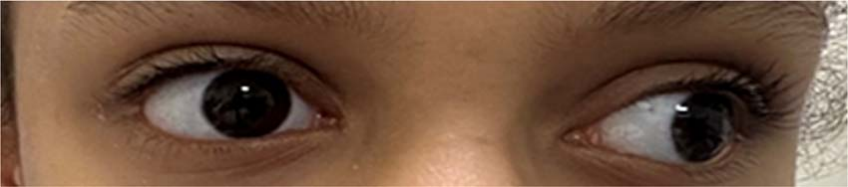
Preoperative image with the patient looking at distance.

The left eye’s Aided Snellen visual acuity was 20/30 at distance and 20/25 at near. Pupils were round and reactive to light and accommodation, and extraocular movements were full in both eyes. No defects were found during color vision screening using the Ishihara S test. Stereopsis, measured with the Titmus test, was 100 arc s. External examination revealed an IXT that was more frequent at distance and was observed less than 50% of the time during a 10-s observation period (Fig. 1).

**Figure 2:**
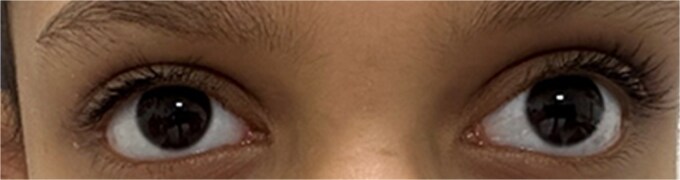
Preoperative image with the patient looking near.

Three measurements on average confirmed the prism and alternate cover test’s (PACT) measurements, which showed 30 prism diopters (PDs) of exodeviation at a distance of 20 feet and 12 PD at a close distance of 40 cm, as shown in Fig. 2.

Fusional response was demonstrated by the Worth Four Dot test at both near and far distances. The results of non-cycloplegic refraction showed that the left eye had +2.00 sphere −2.00 cylinder and the right eye had +3.00 sphere −3.00 cylinder. The digitally measured intraocular pressures were equal and normal on both sides, and the anterior segment examination revealed nothing unusual. After administering two drops of 1% cyclopentolate hydrochloride five minutes apart, cycloplegic retinoscopy revealed +3.75–3.50 × 180 in the right eye and +2.75–2.75 × 180 in the left. A dilated fundus examination showed bilaterally normal peripheral retinae and a cup-to-disc ratio of 0.35 in each eye. Table 1 summarizes the clinical findings from all visits.

**Table 1:** Summary of clinical findings across visits for patient R

**Parameter**	**Measurement**
**Initial visit**	
Visual acuity (aided, Snellen)	OD: 20/30, OS: 20/25 (distance and near) extraocular movements full bilaterally
Pupils	Round, reactive to light and color vision (Ishihara’s test) no defects
Stereopsis (Titmus test, near)	100 arc s
Exotropia observation	Intermittent at distance, <50% of time (10 s) PACT (distance, 20 ft) 30 PD exodeviation
PACT (near, 40 cm)	12 PD exodeviation
Worth four dot test	Fusional response at distance and near
Non-cycloplegic refraction	OD: +3.00–3.00 × 180, OS: +2.00–2.00 × 180
Cycloplegic refraction	OD: +3.75–3.50 × 180, OS: +2.75–2.75 × 180 anterior segment unremarkable
Intraocular pressure	Soft, equal bilaterally (digital)
Fundus exam	Cup-to-disc ratio 0.35 OU, normal peripheral retinae
**Visit 2**	
Mayo scale control	Score 3 (<50% manifestation, 30 s observation)
Surgical intervention	
Procedure	Bilateral lateral rectus recession (7 mm), bilateral medial rectus Faden
Postoperative Day 1	
PACT (distance and near)	Orthophoria
Follow-up (3 and 6 months)	
Without glasses	Distance: 5 PD exotropia, near: orthophoria
With glasses	Distance and near: orthophoria

### Extended evaluation

An extended IXT evaluation was performed at the second visit to assess control and to use the Mayo scale [5] to quantify the deviation. Exotropia was observed for less than 50% of the time during a 30-s observation period; this corresponds to a score of 3, which indicates moderate control.

### Surgical intervention

Surgical intervention was planned given the accurate DE diagnosis, characterized by a high AC/A ratio and a 30 PD distance versus 12 PD near exodeviation. The mean deviation (17 PD) was calculated as the average of the distance and near measurements. Bilateral lateral rectus recession (7 mm) was performed to address the distance deviation. The bilateral medial rectus Faden procedure was used to control the near deviation, considering the potential need for postoperative bifocal glasses [5].

### Postoperative outcomes

**Figure 3:**
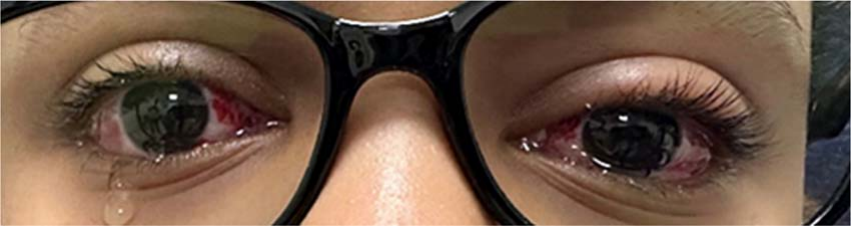
First day postoperative image with the patient looking at distance (orthophoria with alternate prism and cover test).

**Figure 4:**
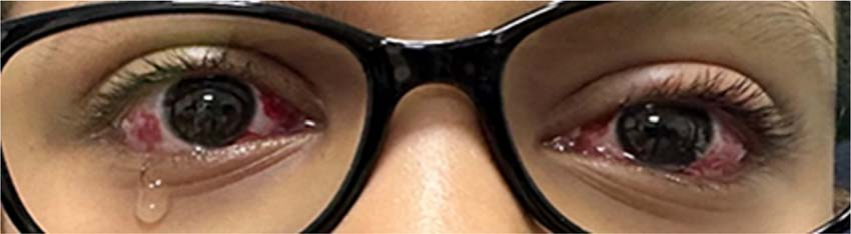
First day postoperative image with the patient looking at near (orthophoria with alternate prism and cover test).

On the first postoperative day, PACT revealed orthophoria at both distance and near (Figs 3 and 4). Follow-up visits at 3 and 6 months showed a slight residual exotropia (5 PD) at distance without glasses, with orthophoria at near. With glasses, the patient achieved orthophoria at both distance and near.

## Discussion

IXT poses diagnostic and management challenges due to its variable presentation and sporadic nature [2]. True DE, as seen in this case, is characterized by a larger exodeviation at distance than at near, often linked to a high AC/A ratio [4]. Non-surgical treatments, including observation, patching, overminus lens therapy, prisms, and vision therapy are commonly considered [5]. However, surgical intervention is often required for significant deviations or poor control.

In this case, the decision to perform bilateral lateral rectus recession (7 mm) targeted the distance deviation (30 PD), while the bilateral medial rectus Faden procedure addressed the near deviation (12 PD). This combined approach was chosen to mitigate the risk of persistent esotropia at near, a known complication in true DE due to the high AC/A ratio [6]. The mean deviation (17 PD) guided the surgical plan, aligning with recommendations to balance distance and near corrections. The postoperative outcome, orthophoria at distance on Day 1 (Fig. 3) and orthotropic at near (Fig. 4), was stable at 3 and 6 months (with minimal residual exotropia at distance of 5 PDs of exophoria without glasses), which supports the efficacy of this approach. Previous studies reported surgical success rates of 56%–61% for IXT, defined as postoperative deviation of less than 10 PD [5]. This case achieved a deviation of 5 PD at distance without glasses, which falls within these high success criteria.

Due to the high AC/A ratio, the use of bifocal glasses postoperatively was considered; however, the patient achieved orthophoria with standard correction. This suggests the Faden procedure effectively controlled the near deviation. The case underscores the importance of tailoring surgical plans to the specific characteristics of DE, considering both distance and near deviations and the potential for postoperative esotropia.

## Conclusion

This case report demonstrates the successful surgical management of true DE exotropia in a 9-year-old patient with IXT. Bilateral lateral rectus recession combined with medial rectus Faden procedure effectively addressed the distance and near deviations, achieving orthophoria postoperatively and stable outcomes at 3 and 6 months. The findings highlight the importance of individualized treatment plans, accounting for the high AC/A ratio in true DE and the potential need for bifocal glasses. Clinicians must consider patient-specific factors, including the magnitude of deviation, age, and compliance, when selecting treatment modalities for IXT.

## Data Availability

All relevant data are included in this published article. Additional data can be obtained upon reasonable request to the corresponding author.
